# Supplementary material for: Tox4 regulates transcriptional elongation and reinitiation during murine T cell development
Source: Commun Biol. 2023 Jun 7;6:613. doi: 10.1038/s42003-023-04992-y (PMC10247741; doi:10.1038/s42003-023-04992-y)
Supplement: Supplementary file 2 — Supplementary Information [file 42003_2023_4992_MOESM2_ESM.pdf]

Supplementary Tables

Supplementary Table 1 Antibodies for FACS.

Target	Fluor	Vender	Catalogue number	Applications
**Lin**	eFluor 450	ebiosciences	88-7772-72	FACS
**C-kit**	SB 600	ebiosciences	63-1171-82	FACS
**Sca-1**	PE-Cy7	ebiosciences	25-5981-82	FACS
**CD150**	FITC	ebiosciences	11-1502-82	FACS
**CD48**	APC	ebiosciences	17-0481-82	FACS
**Annexin V**	FITC	ebiosciences	BMS147FI	FACS
**Ki-67**	APC	ebiosciences	17-5698-82	FACS
**CD3**	eFluro 450	ebiosciences	48-0032-82	FACS
**CD4**	PE-Cy7	ebiosciences	25-0041-81	FACS
**CD8**	FITC	ebiosciences	11-0081-82	FACS
**CD44**	APC	ebiosciences	17-0441-82	FACS
**CD25**	APC-e780	ebiosciences	47-0251-82	FACS
**CD25**	SB 600	ebiosciences	63-0251-82	FACS
**CD69**	APC-e780	ebiosciences	47-0691-82	FACS
**CD8**	APC-e780	ebiosciences	47-0081-82	FACS
**B220**	FITC	ebiosciences	11-0452-82	FACS
**CD19**	APC-e780	ebiosciences	47-0193-82	FACS
**IgM**	APC	ebiosciences	17-5790-82	FACS
**CD43**	PE	ebiosciences	12-0431-82	FACS
**CD24**	APC-e780	ebiosciences	47-0242-82	FACS
**CD71**	FITC	ebiosciences	11-0711-82	FACS
**Ter119**	PE	ebiosciences	12-5921-82	FACS
**CD11b**	FITC	ebiosciences	11-0112-82	FACS
**Gr1**	PE	ebiosciences	12-9668-82	FACS
**TCRβ**	PE	ebiosciences	12-5961-82	FACS
**CD16/32**		ebiosciences	14-0161-86	FACS
**CD3ε**		Biolegend	100339	FACS
**CD28**		Biolegend	102115	FACS

Supplementary Table 2 Antibodies for WB and CUT&Tag.

Target	Vender	Catalogue number	Applications
**Pol II**	Santa Cruz	sc-899	WB, CUT&Tag
**Pol II Ser-2p**	Active Motif	61083	WB, CUT&Tag
**Pol II Ser-5p**	Active Motif	61085	WB, CUT&Tag
**TOX4**	Bethyl	A304-873A	WB, CUT&Tag
**SPT5**	Bethyl	A300-868A	WB, CUT&Tag
**p-SPT5 Thr806**	Fisher Lab		WB, CUT&Tag

Supplementary Table 3 Primers for qRT-PCR.

Name	Sequence	Note
**Cd8a Forward**	GCCCCAGAGACCAGAAGATT	For qRT-PCR of Cd8a
**Cd8a Reverse**	TCTCTGAAGGTCTGGGCTTG	For qRT-PCR of Cd8a
**Cd8b1 Forward**	GCCAGAGGACAGTGACTTCT	For qRT-PCR of Cd8b1
**Cd8b1 Reverse**	ACGGGCATTGCTTCTTCTTC	For qRT-PCR of Cd8b1
**Cdk1 Forward**	TCCTGGGCAGTTCATGGATT	For qRT-PCR of Cdk1
**Cdk1 Reverse**	GTACCACAGCGTCACTACCT	For qRT-PCR of Cdk1
**Ccnb1 Forward**	TTGTGTGCCCAAGAAGATGC	For qRT-PCR of Ccnb1
**Ccnb1 Reverse**	ACGTCAACCTCTCCGACTTT	For qRT-PCR of Ccnb1
**Ccnb2 Forward**	CACCAAAGTACCAGCTCTGC	For qRT-PCR of Ccnb2
**Ccnb2 Reverse**	GCATCAGAGAAAGCTTGGCA	For qRT-PCR of Ccnb2
**Ccna2 Forward**	CTGTCTCTTTACCCGGAGCA	For qRT-PCR of Ccna2
**Ccna2 Reverse**	ATGTCTGGCTGCCTCTTCAT	For qRT-PCR of Ccna2

Supplementary Figures

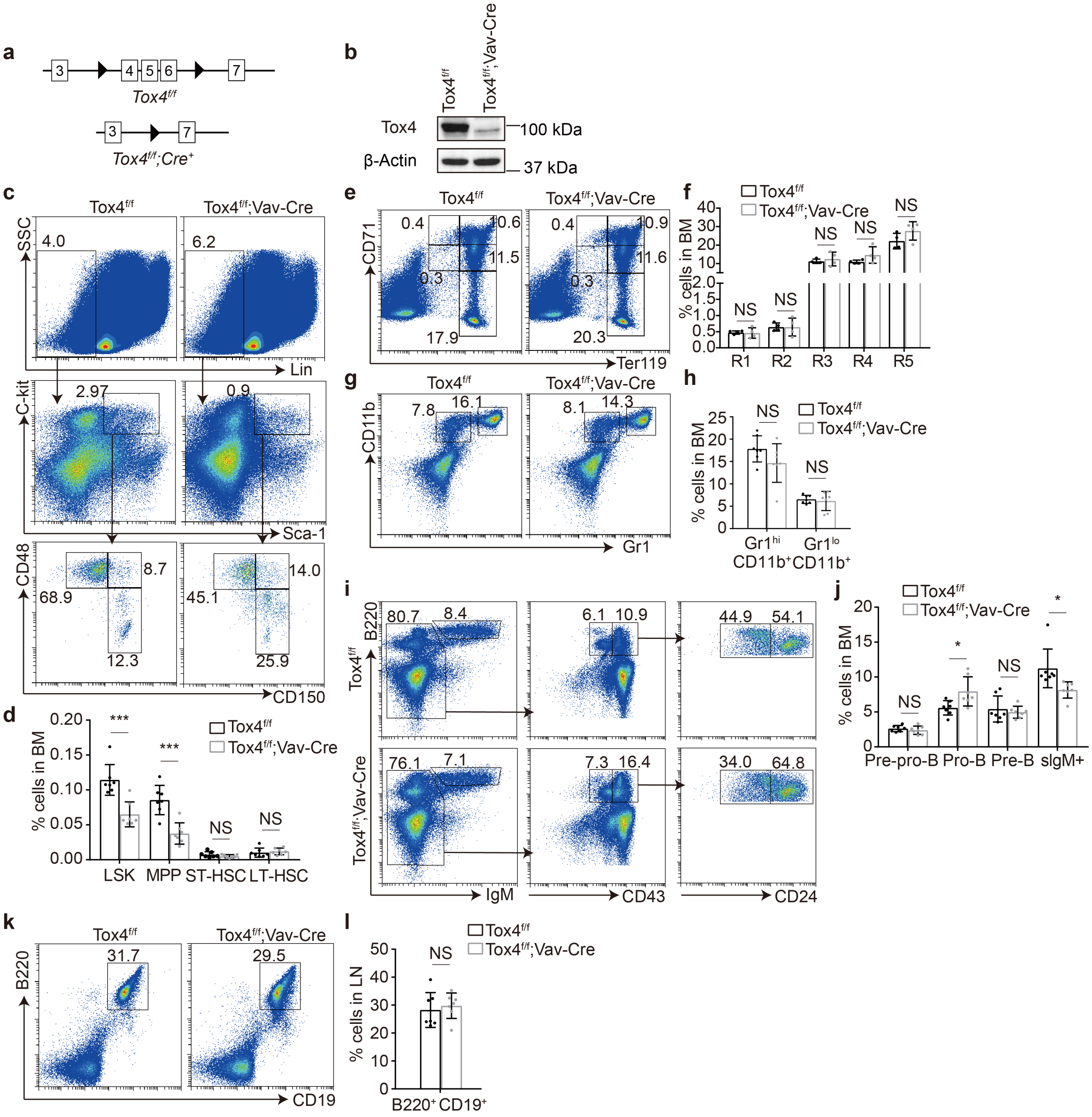

Supplementary Fig. 1 Tox4 deficiency affects the development of multipotential progenitors and T cells.

a Schematic for conditional knockout of *Tox4* using the Cre-loxP system. **b** Determination of the excision efficiency of *Tox4* by Western blot using lysate of thymocytes from *Tox4*$^{f/f}$ (control) and *Tox4*$^{f/f}$*;Vav-Cre* (conditional knockout, cKO) mice. **c** Representative plots of flow cytometric analysis of expression of lineage markers (Lin), c-Kit, Sca-1, CD48 and CD150 in bone marrow

cells. **d** A bar graph comparing frequency of hematopoietic progenitor cells in control and cKO mice. LSK: $Lin^-c\text{-}Kit^+Sca\text{-}1^+$, long-term hematopoietic stem cells (LT-HSC): $Lin^-c\text{-}Kit^+Sca\text{-}1^+CD48^-CD150^+$, short-term hematopoietic stem cells (ST-HSC): $Lin^-c\text{-}Kit^+Sca\text{-}1^+CD48^+CD150^+$, and multipotential progenitors (MPP): $Lin^-c\text{-}Kit^+Sca\text{-}1^+CD48^+CD150^-$. **e** Representative plots of flow cytometric analysis of expression of CD71 and Ter119 in bone marrow cells. **f** A bar graph comparing frequency of erythroid cell subpopulations in control and cKO mice. R1: $CD71^{med}Ter119^{low}$, R2: $CD71^{high}Ter119^{low}$, R3: $CD71^{high}Ter119^{high}$, R4: $CD71^{med}Ter119^{high}$, and R5: $CD71^{low}Ter119^{high}$. **g** Representative plots of flow cytometric analysis of expression of CD11b and Gr1 in bone marrow cells. **h** A bar graph comparing frequency of myeloid cell subpopulations in control and cKO mice. neutrophils: $CD11b^+Gr1^{high}$, and monocytes, macrophages and eosinophiles: $CD11b^+Gr1^{low}$. **i** Representative plots of flow cytometric analysis of expression of B220, IgM, CD43 and CD24 in bone marrow cells. **j** A bar graph comparing frequency of B cell subpopulations in control and cKO mice. Pre-pro-B: $B220^+IgM^-CD43^+CD24^{low}$, Pro-B: $B220^+IgM^-CD43^+CD24^+$, Pre-B: $B220^+IgM^-CD43^-$ and sIgM+: $B220^+IgM^+$. **k** Representative plots of flow cytometric analysis of expression of B220 and CD19 in lymphocytes. **l** A bar graph comparing frequency of lymphatic B cells in control and cKO mice. All mice were analyzed at 6–8 weeks of age. Pictures in **c**, **g**, **i** and **k** are representative of seven independent experiments (n=7). Pictures in **e** are representative of four independent experiments (n=4). Statistical significance was determined with a two-sided Student's t-test; the centers and the error bars represent the mean and the SD, respectively. NS: $P \geq 0.05$, $^*P < 0.05$ and $^{***}P < 0.001$.

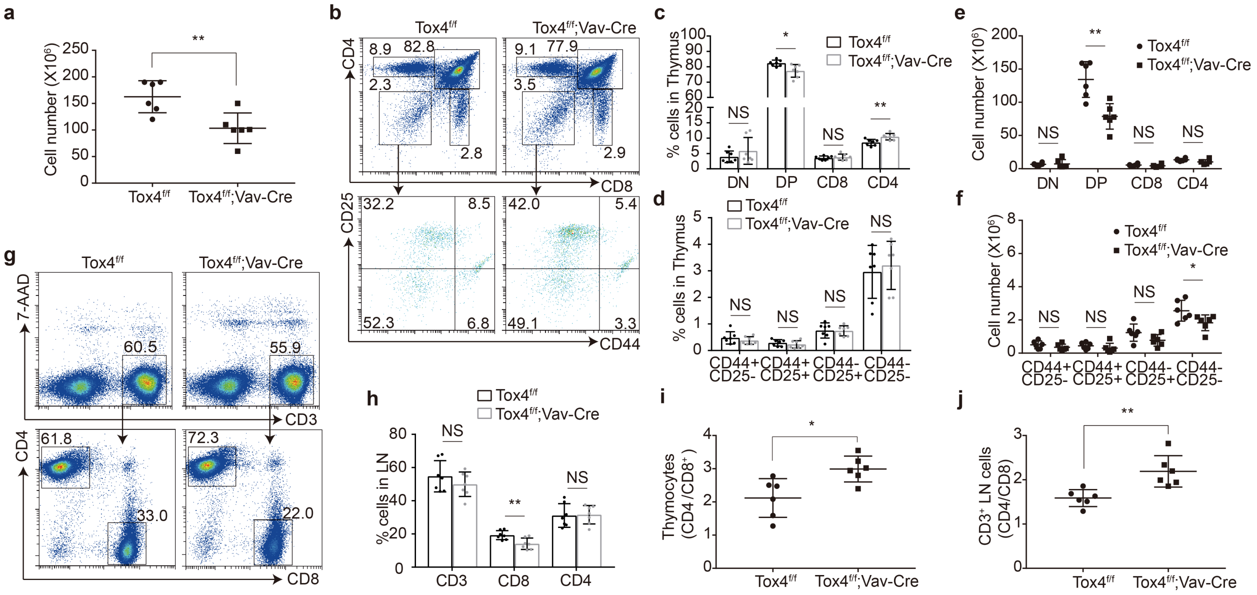

Supplementary Fig. 2 Pan-hematopoietic *Tox4* deletion impairs T cell development.

a A scatter plot comparing thymic cellularity of *Tox4$^{f/f}$* (control) and *Tox4$^{f/f}$;Vav-Cre* (cKO) mice. **b** Representative plots of flow cytometric analysis of expression of CD4, CD8, CD25 and CD44 in thymocytes. **c**, **d** Bar graphs comparing frequency of αβ T cell populations (**c**) and DN1-4 within the DN population (**d**) in control and cKO mice. DN: $CD4^-CD8^-$, DP: $CD4^+CD8^+$, CD4: $CD4^+CD8^-$, CD8: $CD4^-CD8^+$, DN1: $CD4^-CD8^-CD44^+CD25^-$, DN2: $CD4^-CD8^-CD44^+CD25^+$, DN3: $CD4^-CD8^-CD44^-CD25^+$, and DN4: $CD4^-CD8^-CD44^-CD25^-$. **e**, **f** Scatter plots comparing numbers of major αβ T cell populations (**e**) and DN1-4 within the DN population (**f**) in control and cKO mice. **g** Representative plots of flow cytometric analysis of expression of CD3, CD4 and CD8 in lymphocytes. **h** A bar graph comparing frequency of lymphatic T cell populations in control and cKO mice. **i**, **j** Scatter plots comparing ratio of CD4 to $CD8^+$ T cells in the thymus (**i**) and the lymph nodes (**j**) in control and cKO mice. All mice were analyzed at 6–8 weeks of age. Pictures in **b** and **g** are representative of six (n=6) and seven (n=7) independent experiments, respectively. Statistical significance was determined with a two-sided Student's t-test; the centers and the error bars represent the mean and the SD, respectively. NS: $P \geq 0.05$, $^*P < 0.05$ and $^{**}P < 0.01$.

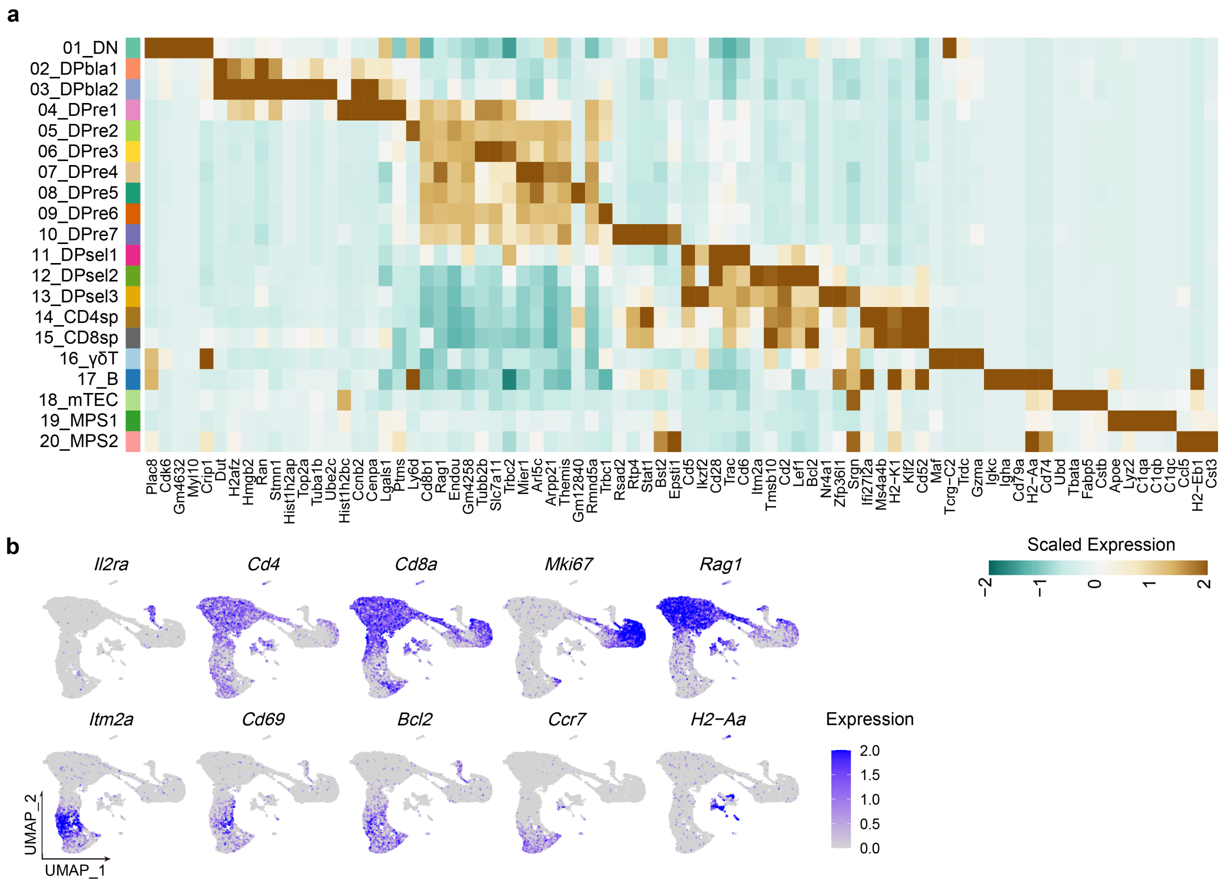

Supplementary Fig. 3 Expression of some of the recognized marker genes in the thymocyte clusters.

a A heatmap presenting scaled mean expression of some of the recognized markers within each cluster of thymocytes. **b** Projection of marker genes *Il2ra*, *Cd4*, *Cd8a*, *Mki67*, *Rag1*, *Itm2a*, *Cd69*, *Bcl2*, *Ccr7* and *H2-Aa* onto UMAP plots. Color bar, normalized expression value.

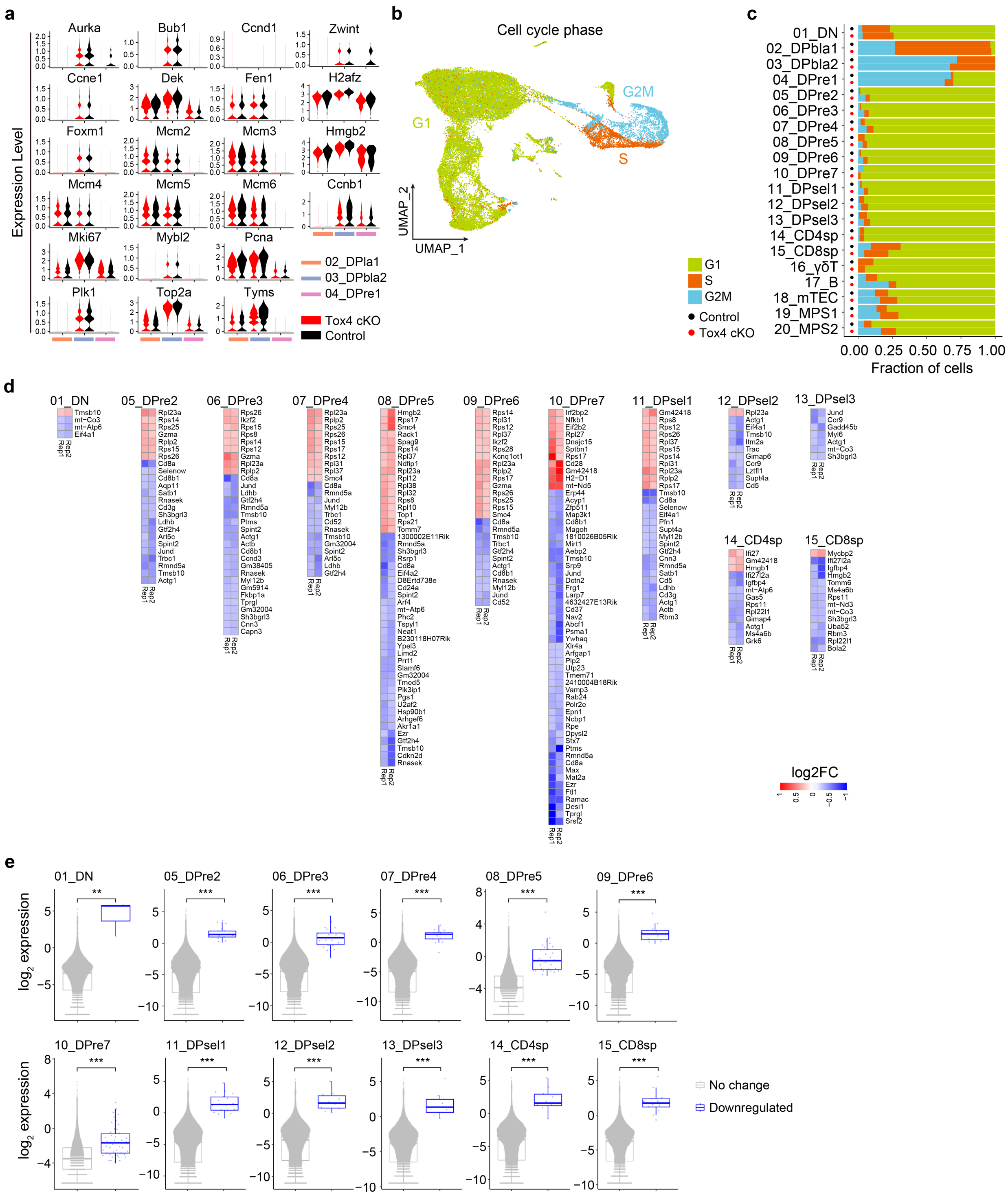
a
Expression Level
Aurka
Bub1
Ccnd1
Zwint
Ccne1
Dek
Fen1
H2afz
Foxm1
Mcm2
Mcm3
Hmgb2
Mcm4
Mcm5
Mcm6
Ccnb1
Mki67
Mybl2
Pcna
Plk1
Top2a
Tyms
02_DPla1
03_DPbla2
04_DPre1
Tox4 cKO
Control
b
Cell cycle phase
G1
G2M
S
UMAP_2
UMAP_1
G1
S
G2M
Control
Tox4 cKO
c
01_DN
02_DPbla1
03_DPbla2
04_DPre1
05_DPre2
06_DPre3
07_DPre4
08_DPre5
09_DPre6
10_DPre7
11_DPsel1
12_DPsel2
13_DPsel3
14_CD4sp
15_CD8sp
16_γδT
17_B
18_mTEC
19_MPS1
20_MPS2
0.00 0.25 0.50 0.75 1.00
Fraction of cells
d
01_DN
05_DPre2
06_DPre3
07_DPre4
08_DPre5
09_DPre6
10_DPre7
11_DPsel1
12_DPsel2
13_DPsel3
14_CD4sp
15_CD8sp
Rep1 Rep2
log2FC
1 0.5 0 -0.5 -1
e
log2 expression
01_DN
05_DPre2
06_DPre3
07_DPre4
08_DPre5
09_DPre6
10_DPre7
11_DPsel1
12_DPsel2
13_DPsel3
14_CD4sp
15_CD8sp
No change
Downregulated

Supplementary Fig. 4 Tox4 loss differentially affects gene expression in thymocyte subpopulations.

a Violin plots comparing normalized expression of selected proliferation-related genes within DPbla1, DPbla2 and DPre1 clusters of thymocytes from control and cKO mice. **b** Combined UMAP plot showing cell cycle phase of thymocytes from control and cKO mice (2 pairs). **c** A bar graph showing frequency of cells in G1, S and G2/M phases of the cell cycle within each cluster of thymocytes of control and cKO mice. **d** Heatmaps showing $\log_2$ fold change of differentially expressed genes in each $\alpha\beta$ T cell cluster upon Tox4 loss excluding those of 02_DPbla1, 03_DPbla2 and 04_DPre1. **e** Violin plots comparing expression level of downregulated and unaffected genes within each $\alpha\beta$ T cell cluster upon Tox4 loss excluding those of 02_DPbla1, 03_DPbla2 and 04_DPre1. Two independent experiments were performed (n=2). Expression differences between two groups of genes were tested using two-sided Wilcoxon Rank Sum test; the standard boxplot notation was used (lower/upper hinges--first/third quartiles; whiskers extend from the hinges to the largest/lowest values no further than 1.5 * inter-quartile ranges; middle line--the median), $^{**}P < 0.01$ and $^{***}P < 0.001$.

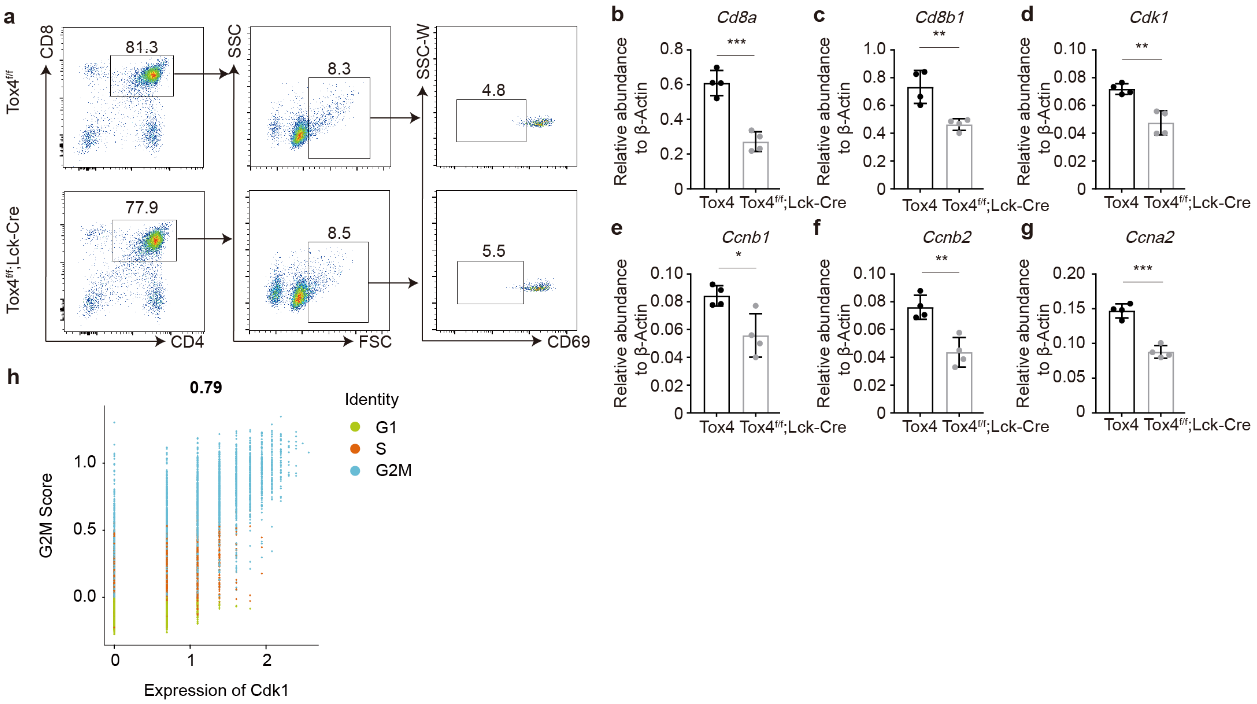

Supplementary Fig. 5 Tox4 regulates transcription of TCR signaling- and the cell cycle-related genes.

a Representative FACS plots of sorting DP blast cells ($CD4^+CD8^+FSC^{hi}CD69^{lo}$) from control and cKO mice. **b-g** Comparison of mRNA level of *Cd8a* (**b**), *Cd8b1* (**c**), *Cdk1* (**d**), *Ccnb1* (**e**), *Ccnb2* (**f**) and *Ccna2* (**g**) by quantitative RT-PCR. Pictures in **b-g** are representative of four independent experiments (n=4). Statistical significance was determined with a two-sided Student's t-test; the centers and the error bars represent the mean and the SD, respectively. *P < 0.05, **P < 0.01 and ***P < 0.001. **h** A scatter plot showing a positive correlation between calculated G2M score and Cdk1 expression. Pearson correlation coefficient is placed on top of the plot. Dots are colored according to the estimated cell cycle phase.

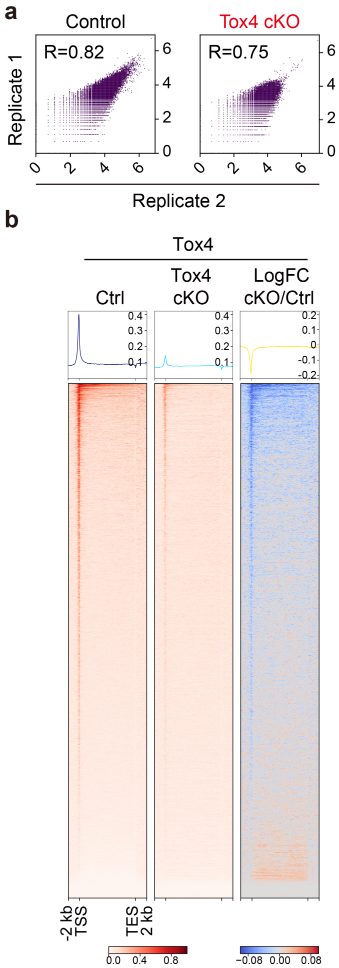

Supplementary Fig. 6 Analyses of chromatin occupancy of Tox4 by CUT&Tag.

a Correlation plots for biological replicates of Tox4 CUT&Tag in control and cKO DP cells. **b** Genome-wide meta-gene profiles and heatmaps of CUT&Tag comparing chromatin occupancy of Tox4 in cKO versus control cells.

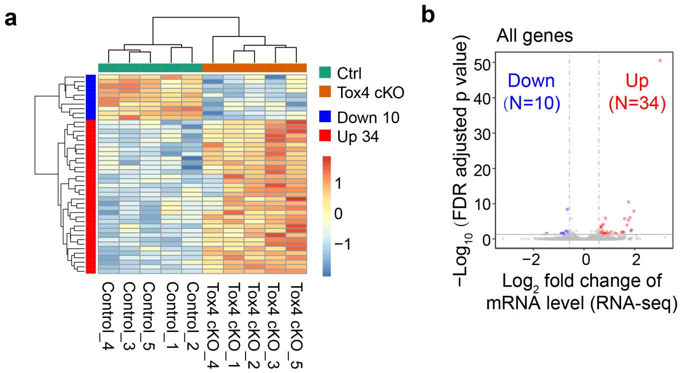

Supplementary Fig. 7 Tox4 deficiency affects expression of a small subset of genes in CD8 thymocytes.

a A heatmap comparing expression of significantly affected genes in Tox4 cKO versus control cells. **b** A volcano plot comparing mRNA level changes of genes in cKO versus control thymocytes.

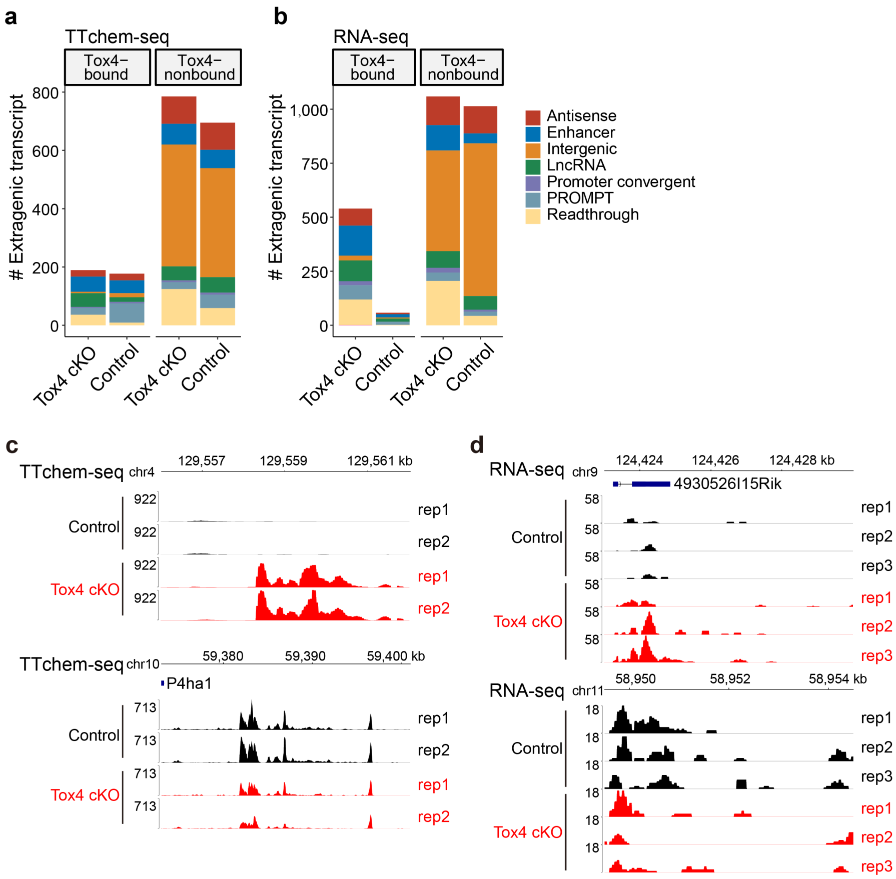

Supplementary Fig. 8 Tox4 regulates expression of a small subset of extragenic transcripts in DP cells.

a A stacked bar graph showing annotation of significantly upregulated Tox4 bound (left) and Tox4 nonbound (right) extragenic transcripts in cKO and control cells, respectively, identified by CUT&Tag and TTchem-seq. Note that significantly upregulated ones in control are downregulated in cKO. **b** A stacked bar graph showing annotation of significantly upregulated Tox4 bound (left) and Tox4 nonbound (right) extragenic transcripts in cKO and control cells, respectively, identified by CUT&Tag and RNA-seq. Note that significantly upregulated ones in control are downregulated in cKO. **c** Normalized read distribution of TTchem-seq within two extragenic loci directly regulated by Tox4 in cKO versus control cells. **d** Normalized read distribution of RNA-seq within two extragenic loci directly regulated by Tox4 in cKO versus control cells.

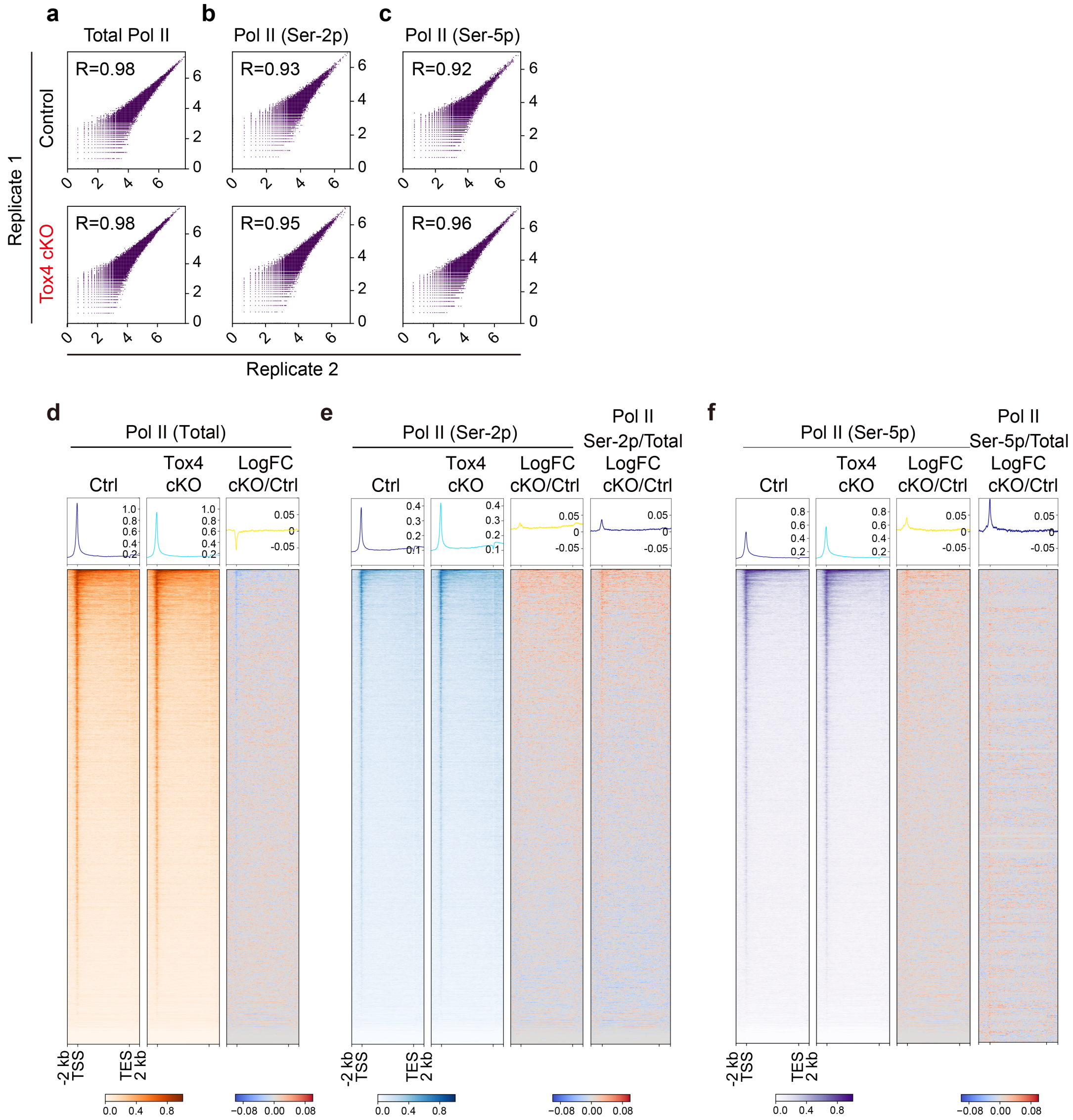

Supplementary Fig. 9 Analyses of chromatin occupancy of total, Ser-2 phosphorylated and Ser-5 phosphorylated Pol II by CUT&Tag.

a, **b**, **c** Correlation plots for biological replicates of total (**a**), Ser-2 phosphorylated (**b**) and Ser-5 phosphorylated (**c**) Pol II CUT&Tag in control and cKO DP cells. **d**, **e**, **f** Genome-wide meta-gene profiles and heatmaps of CUT&Tag comparing chromatin occupancy of total (**d**), Ser-2 phosphorylated (**e**) and Ser-5 phosphorylated (**f**) Pol II in cKO versus control cells.

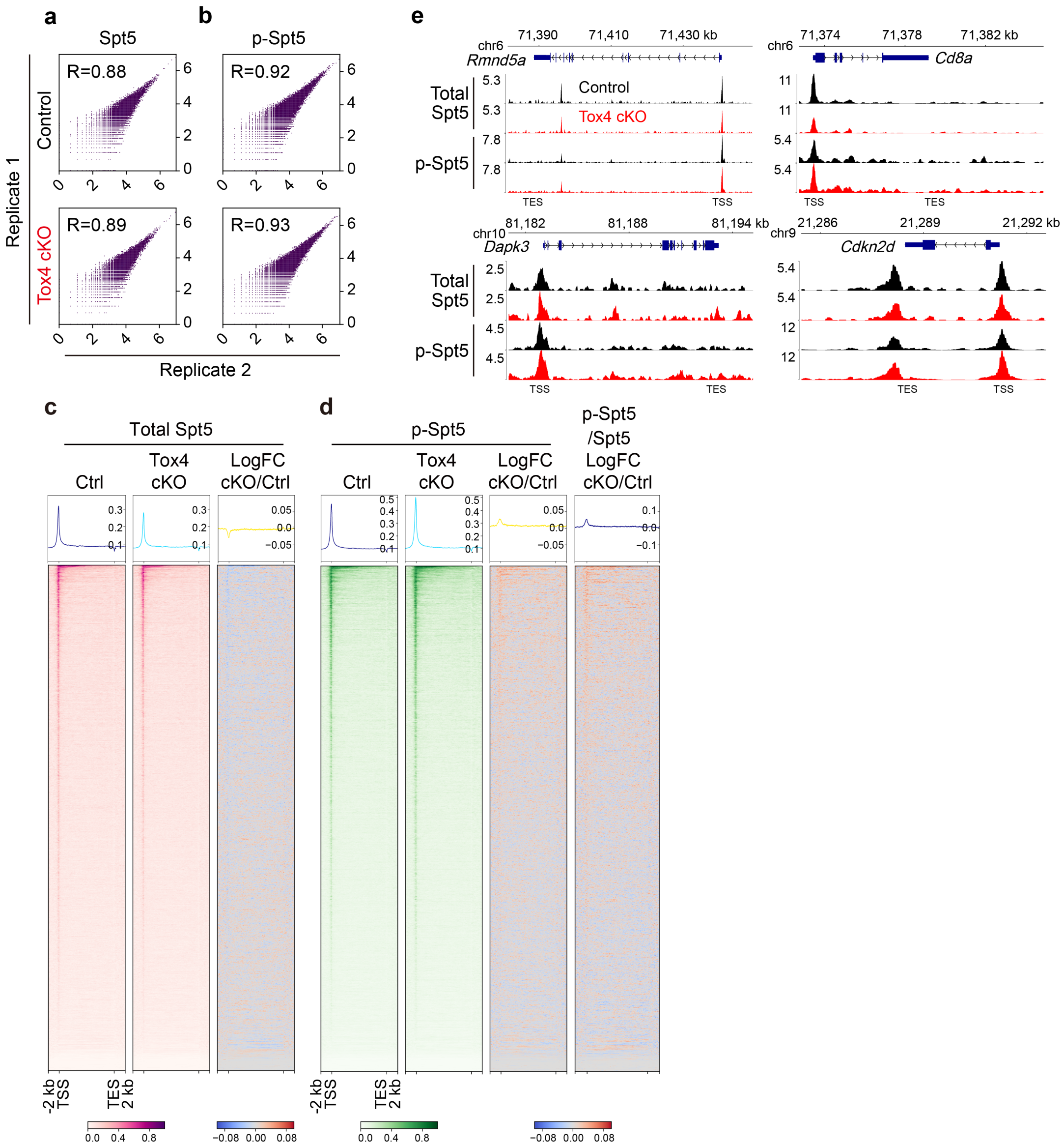

Supplementary Fig. 10 Analyses of chromatin occupancy of Spt5 and p-Spt5 Thr-806 by CUT&Tag.

a, **b** Correlation plots for biological replicates of Spt5 (**a**) and p-Spt5 Thr806 (**b**) CUT&Tag in control and cKO DP cells. **c**, **d** Genome-wide meta-gene profiles and heatmaps of CUT&Tag comparing chromatin occupancy of total Spt5 (**c**) and p-Spt5 Thr-806 (**d**) in cKO versus control cells. **e** Normalized read distribution of CUT&Tag of Spt5 and p-Spt5 Thr-806 within the *Rmnd5a*, *Cd8a*, *Dapk3* and *Cdkn2d* loci in cKO versus control cells.

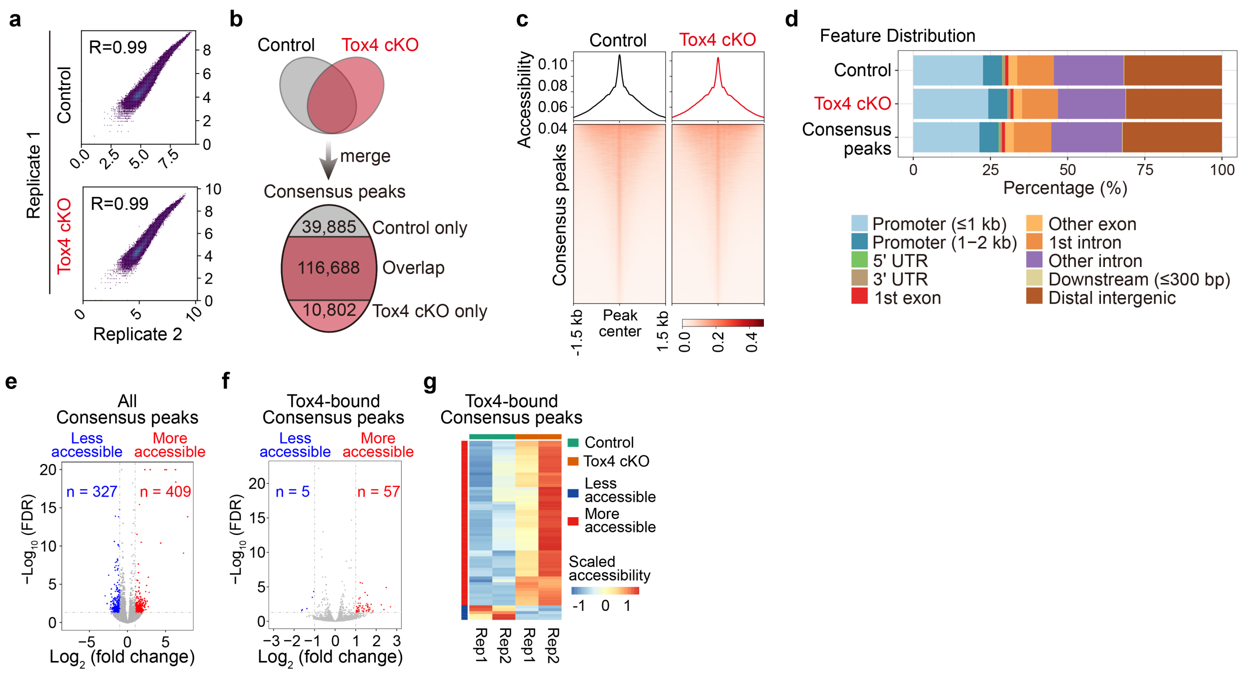

Supplementary Fig. 11 Tox4 loss affects chromatin accessibility of a small subset of genes.

a Correlation plots for biological replicates of ATAC-seq in control (top) and cKO (bottom) DP cells. **b** A diagram describing the obtainment of consensus peaks by merging peaks of control and cKO DP cells. **c** Chromatin accessibility near consensus peak center in control and cKO DP cells. **d** Genomic annotation of peaks in control and cKO DP cells and the consensus peaks. **e**, **f** Volcano plots showing chromatin accessibility changes (**e**) and chromatin accessibility changes of Tox4 binding sites (**f**) upon Tox4 loss in DP cells. **g** A heatmap showing Tox4 binding sites with significant accessibility change in cKO versus control DP cells.

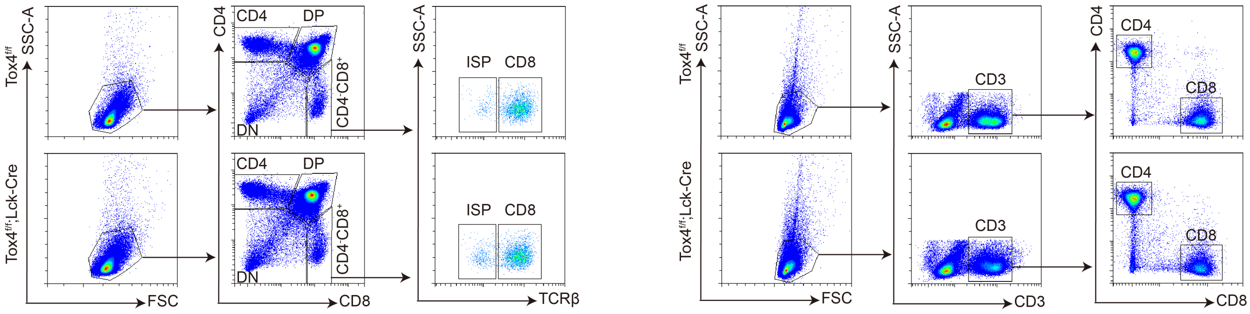

Supplementary Fig. 12 Gating strategy of FACS referring to Fig. 2a and c.

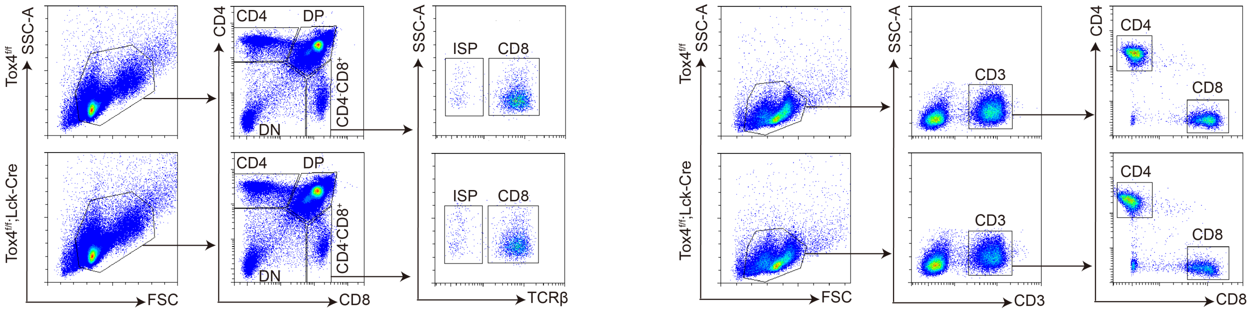

Supplementary Fig. 13 Gating strategy of FACS referring to Fig. 2e and h.

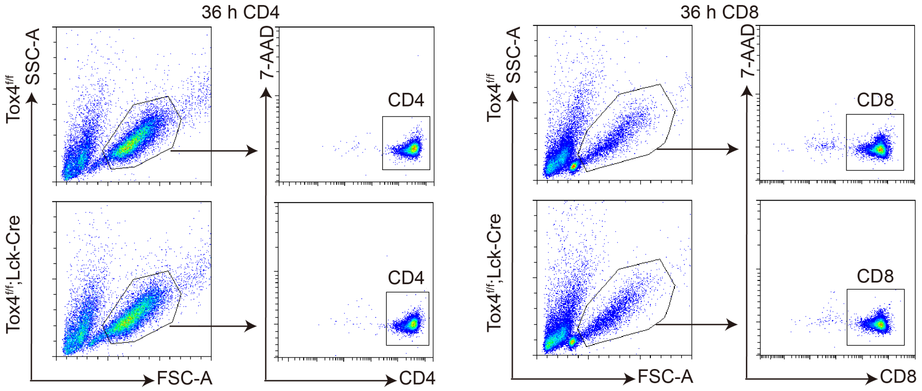

Supplementary Fig. 14 Gating strategy of FACS referring to Fig. 3a, b, f and g.

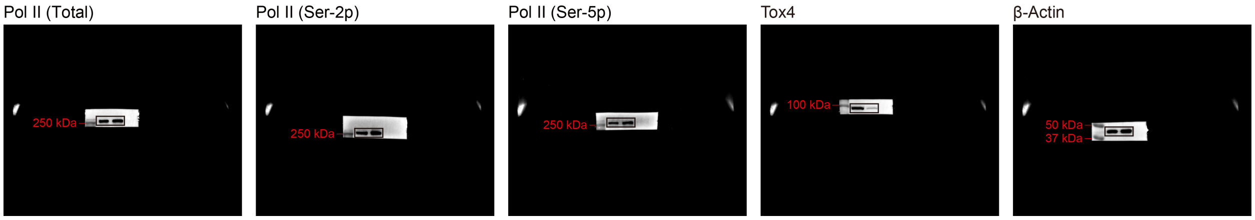

Supplementary Fig. 15 Uncropped blots referring to Fig. 1b.

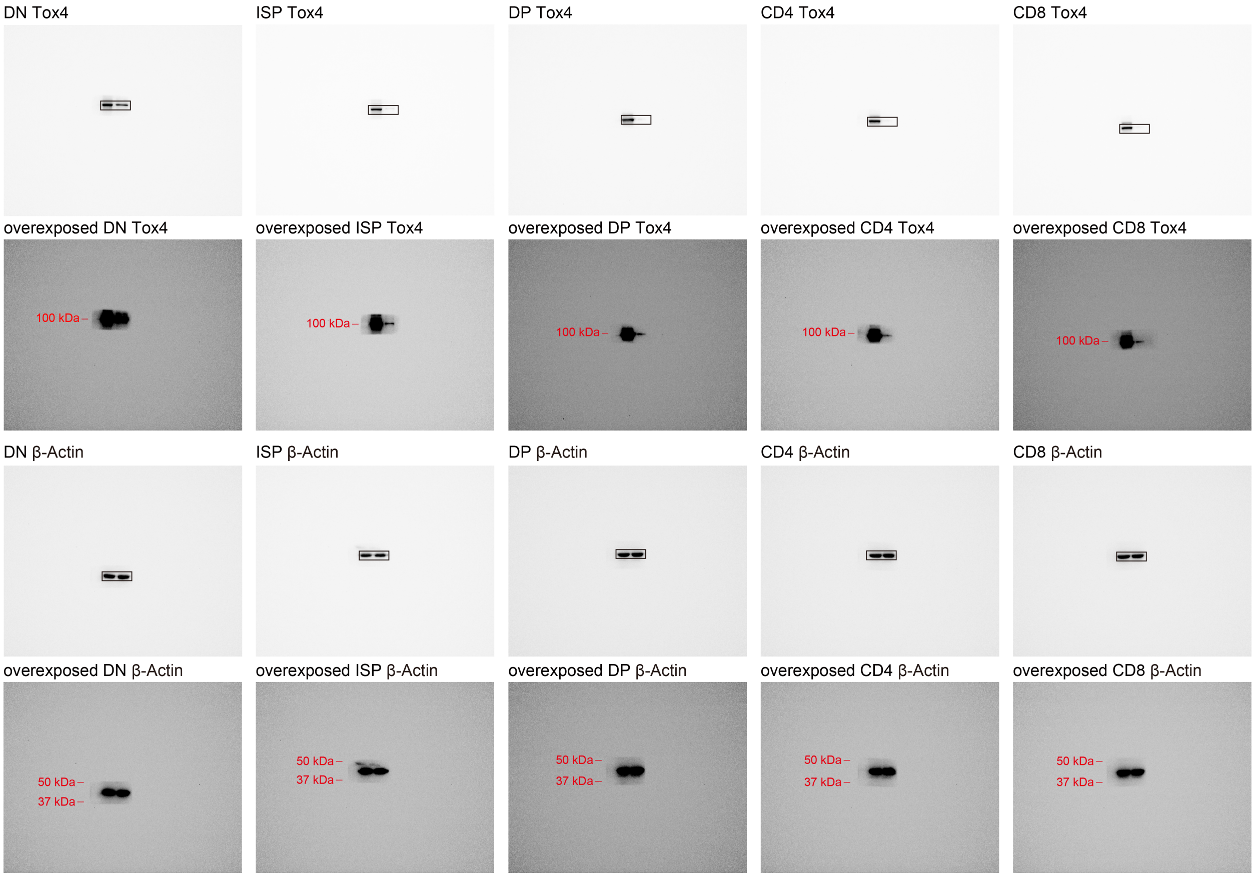

Supplementary Fig. 16 Uncropped blots referring to Fig. 1c.

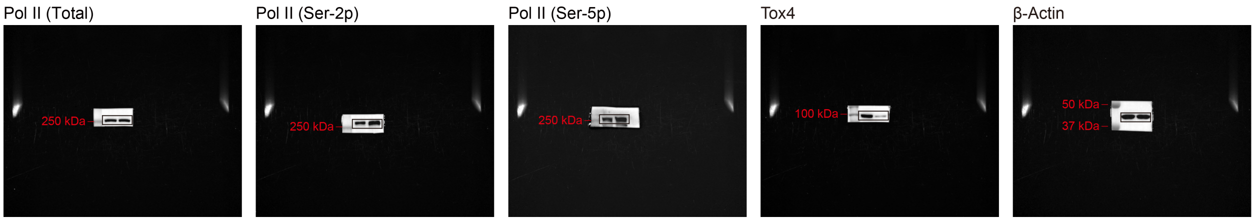

Supplementary Fig. 17 Uncropped blots referring to Fig. 6k.

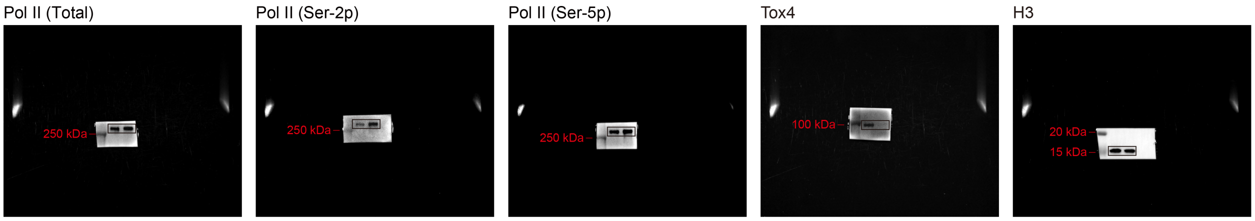

Supplementary Fig. 18 Uncropped blots referring to Fig. 6l.

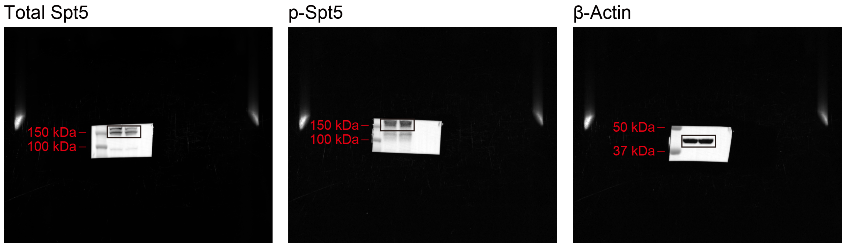

Supplementary Fig. 19 Uncropped blots referring to Fig. 7a.

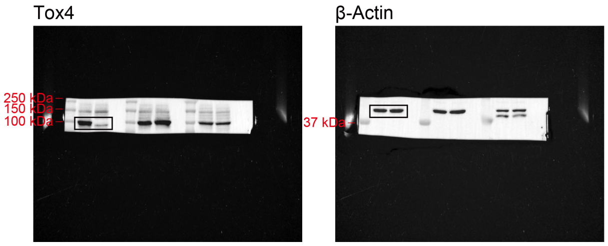

Supplementary Fig. 20 Uncropped blots referring to Supplementary Fig. 1b.
