# Supplementary material for: Tox4 regulates transcriptional elongation and reinitiation during murine T cell development
Source: Commun Biol. 2023 Jun 7;6:613. doi: 10.1038/s42003-023-04992-y (PMC10247741; doi:10.1038/s42003-023-04992-y)
Supplement: Supplementary file 5 — Reporting Summary [file 42003_2023_4992_MOESM5_ESM.pdf]

nature portfolio

Corresponding author(s): Ming Yu

Last updated by author(s): May 18, 2023

Reporting Summary

Nature Portfolio wishes to improve the reproducibility of the work that we publish. This form provides structure for consistency and transparency in reporting. For further information on Nature Portfolio policies, see our Editorial Policies and the Editorial Policy Checklist.

Statistics

For all statistical analyses, confirm that the following items are present in the figure legend, table legend, main text, or Methods section.

n/a	Confirmed	
☐	☒	The exact sample size (*n*) for each experimental group/condition, given as a discrete number and unit of measurement
☐	☒	A statement on whether measurements were taken from distinct samples or whether the same sample was measured repeatedly
☐	☒	The statistical test(s) used AND whether they are one- or two-sided<br>*Only common tests should be described solely by name; describe more complex techniques in the Methods section.*
☒	☐	A description of all covariates tested
☐	☒	A description of any assumptions or corrections, such as tests of normality and adjustment for multiple comparisons
☐	☒	A full description of the statistical parameters including central tendency (e.g. means) or other basic estimates (e.g. regression coefficient) AND variation (e.g. standard deviation) or associated estimates of uncertainty (e.g. confidence intervals)
☐	☒	For null hypothesis testing, the test statistic (e.g. *F*, *t*, *r*) with confidence intervals, effect sizes, degrees of freedom and *P* value noted<br>*Give P values as exact values whenever suitable.*
☒	☐	For Bayesian analysis, information on the choice of priors and Markov chain Monte Carlo settings
☒	☐	For hierarchical and complex designs, identification of the appropriate level for tests and full reporting of outcomes
☐	☒	Estimates of effect sizes (e.g. Cohen's *d*, Pearson's *r*), indicating how they were calculated

Our web collection on statistics for biologists contains articles on many of the points above.

Software and code

Policy information about availability of computer code

Data collection	No software was used for the data collection.
Data analysis	CUT&Tag<br>Raw reads were filtered using fastp (v0.13.1) and subsequently aligned to the human genome using Bowtie2 (v2.3.4.1). Aligned reads were filtered using Samtools (v1.9) and duplicates were removed with Picard. For CUT&Tag, peak calling was performed using SEACR (v1.3), and for ChIP-seq, peak calling was performed using MACS2 (v2.2.6). Annotation of peaks was performed using ChIPseeker (v1.18.0).<br><br>TT-seq<br>Raw reads were filtered using fastp (v0.13.1) and subsequently aligned to the human genome and the yeast genome using STAR (v2.7.9a), respectively. SAMtools (v0.1.19) and Picard were used to sort, index and mark duplicate reads in the resulting genome BAM files. Scale factor for each sample were estimated, and the differential expression analysis was performed with DESeq2 (v1.22.2). Strand-specific metagene profiles were generated with Ngs.plot.<br><br>RNA-seq<br>Raw reads were filtered using fastp (v0.13.1) and subsequently aligned to the human genome and the yeast genome using HISAT2 (v2.1.0). Gene level read count were calculated using featureCounts (v1.6.1). The differential expression analysis was performed with DESeq2 (v1.22.2). KEGG pathway enrichment analyses were performed with clusterProfiler (v3.10.1).<br><br>Read count quantification<br>Reproducibility of two biological replicates were assessed using Pearson correlation coefficient calculated by deepTools (v3.3.1). BamCoverage from deepTools was used to generate bigwig files of normalized read coverage per 50-bp bin for CUT&Tag, ChIP-seq, 4sUDRB-seq and TT-seq.

Metagene profile plots were generated using computeMatrix and plotProfile from deepTools unless otherwise stated.

scRNA-seq analyses
scRNA-seq reads were aligned to the GRCm38 (mm10) reference genome and quantified using 'cellranger count' (10x Genomics, version 6.1.1) with default parameters. scRNA-seq UMI count matrices were imported to R 4.1.0 and gene expression data analysis was performed using the R/Seurat package

For manuscripts utilizing custom algorithms or software that are central to the research but not yet described in published literature, software must be made available to editors and reviewers. We strongly encourage code deposition in a community repository (e.g. GitHub). See the Nature Portfolio guidelines for submitting code & software for further information.

Data

Policy information about availability of data

All manuscripts must include a data availability statement. This statement should provide the following information, where applicable:

- Accession codes, unique identifiers, or web links for publicly available datasets
- A description of any restrictions on data availability
- For clinical datasets or third party data, please ensure that the statement adheres to our policy

Next generation sequencing data have been submitted to GEO repository under accession number GSE190041 (the secure token: olyniksavvupbon).

Field-specific reporting

Please select the one below that is the best fit for your research. If you are not sure, read the appropriate sections before making your selection.

[x] Life sciences [ ] Behavioural & social sciences [ ] Ecological, evolutionary & environmental sciences

For a reference copy of the document with all sections, see nature.com/documents/nr-reporting-summary-flat.pdf

Life sciences study design

All studies must disclose on these points even when the disclosure is negative.

Sample size	No statistical methods were used to predetermine sample size.
Data exclusions	No used in this study.
Replication	Two biological replicates were generated initially for each genomic analysis. Two more biological replicates were added if discrepancies were found between the original two biological replicates.
Randomization	Experiments were not randomized.
Blinding	Not used in this study.

Reporting for specific materials, systems and methods

We require information from authors about some types of materials, experimental systems and methods used in many studies. Here, indicate whether each material, system or method listed is relevant to your study. If you are not sure if a list item applies to your research, read the appropriate section before selecting a response.

Materials & experimental systems

n/a	Involved in the study	
[ ]	[x]	Antibodies
[x]	[ ]	Eukaryotic cell lines
[x]	[ ]	Palaeontology and archaeology
[ ]	[x]	Animals and other organisms
[x]	[ ]	Human research participants
[x]	[ ]	Clinical data
[x]	[ ]	Dual use research of concern

Methods

n/a	Involved in the study	
[x]	[ ]	ChIP-seq
[ ]	[x]	Flow cytometry
[x]	[ ]	MRI-based neuroimaging

Antibodies

Antibodies used	Pol II, Santa Cruz, sc-899; Pol II (Ser-2p), clone 3E10, Active Motif, 61083, lot 32418002; Pol II (Ser-5p), clone 3E8, Active Motif, 61085, lot 10618002;TOX4, Bethyl, A304-873A, lot 1; SPT5, Bethyl, A300-868A, lot 1; β-Actin, Santa Cruz, sc-47778, E0720. Annexin V-FITC, eBiosciences, BMS147FI, lot 162889000; Ki-67-APC, eBiosciences, 17-5698-82, lot 4342181; CD3-eFluro 450,

eBiosciences, 48-0032-82, lot 1987699; CD4-PE-Cy7, eBiosciences, 25-0041-81, lot 2123767; CD8-FITC, eBiosciences, 11-0081-82, lot 2213264; CD44-APC, eBiosciences, 17-0441-82, lot 2190983; CD25-APC-e780, eBiosciences, 47-0251-82, lot 2114190;CD25-Super Bright 600, eBiosciences, 63-0251-82, lot 2196770; CD69-APC-e780, eBiosciences, 47-0691-82, lot 2002695;CD8-APC-e780, eBiosciences, 47-0081-82, lot 2011697; CD16/32, eBiosciences, 14-0161-86, lot 2058684; Lin-eFluro 450, eBiosciences, 88-7772-72, lot 4343378; C-kit-SB 600, eBiosciences, 63-1171-82, lot 4337283; Sca1-PE-Cy7, eBiosciences, 25-5981-82, lot 4323278; CD150-FITC, eBiosciences, 11-1502-82, lot 1995312; CD48-APC, eBiosciences, 17-0481-82, lot 4330244; B220-FITC, eBiosciences, 11-0452-82, lot 4290687; CD19-APC-e780, eBiosciences, 47-0193-82, lot 1956871; IgM-APC, eBiosciences, 17-5790-82, lot 1923165; CD43-PE, eBiosciences, 12-0431-82, lot 1942079; CD24-APC-e780, eBiosciences, 47-0242-82, lot 1996371; CD71-FITC, eBiosciences, 11-0711-82, lot 4348372; Ter119-PE, eBiosciences, 12-5921-82, lot 4347833; CD11b-FITC, eBiosciences, 11-0112-82, lot 4332602; Gr1-PE, eBiosciences, 12-9668-82, lot 1923430; Ultra-LEAF™ Purified anti-mouse CD3ε Antibody, Biolegend, Clone 145-2C11, 100339, lot B328304; Ultra-LEAF™ Purified anti-mouse CD28 Antibody, Biolegend, Clone 37.51, 102115, lot B351408; TCRβ-PE, eBiosciences, 12-5961-82, lot 43229907.

Validation

All the antibodies were validated by either the vendor or the contributor.
Pol II(Santa Cruz): https://www.citeab.com/antibodies/825104-sc-899-pol-ii-n-20
Pol II (Ser-2p): https://www.activemotif.com.cn/catalog/details/61083/rna-pol-ii-ctd-phospho-ser2-antibody-mab
Pol II (Ser-5p): https://www.activemotif.com.cn/catalog/details/61085/rna-pol-ii-ctd-phospho-ser5-antibody-mab
TOX4: https://www.bethyl.com/product/A304-873A/TOX4+Antibody
SPT5: https://www.bethyl.com/product/A300-868A/SUPT5H+Antibody
β-Actin: https://www.scbt.com/p/beta-actin-antibody-c4?requestFrom=search
Annexin V-FITC: https://www.thermofisher.cn/cn/zh/antibody/product/Annexin-V-Antibody-clone-VAA-33-Monoclonal/BMS147FI
Ki-67-APC: https://www.thermofisher.cn/cn/zh/antibody/product/Ki-67-Antibody-clone-SolA15-Monoclonal/17-5698-82
CD3-eFluro 450: https://www.thermofisher.cn/cn/zh/antibody/product/CD3-Antibody-clone-17A2-Monoclonal/48-0032-82
CD4-PE-Cy7: https://www.thermofisher.cn/cn/zh/antibody/product/CD4-Antibody-clone-GK1-5-Monoclonal/25-0041-81
CD8-FITC: https://www.thermofisher.cn/cn/zh/antibody/product/CD8a-Antibody-clone-53-6-7-Monoclonal/11-0081-82
CD44-APC: https://www.thermofisher.cn/cn/zh/antibody/product/CD44-Antibody-clone-IM7-Monoclonal/17-0441-82
CD25-APC-e780: https://www.thermofisher.cn/cn/zh/antibody/product/CD25-Antibody-clone-PC61-5-Monoclonal/47-0251-82
CD25-SB 600: https://www.thermofisher.cn/cn/zh/antibody/product/CD25-Antibody-clone-PC61-5-Monoclonal/63-0251-82
CD69-APC-e780: https://www.thermofisher.cn/cn/zh/antibody/product/CD69-Antibody-clone-H1-2F3-Monoclonal/47-0691-82
CD8-APC-e780: https://www.thermofisher.cn/cn/zh/antibody/product/CD8a-Antibody-clone-53-6-7-Monoclonal/47-0081-82
CD16/32: https://www.thermofisher.cn/cn/zh/antibody/product/CD16-CD32-Antibody-clone-93-Monoclonal/14-0161-86
Lin-eFluro 450: https://www.thermofisher.cn/cn/zh/antibody/product/Mouse-Hematopoietic-Lineage-Antibody-Cocktail/88-7772-72
C-kit-SB 600: https://www.thermofisher.cn/cn/zh/antibody/product/CD117-c-Kit-Antibody-clone-2B8-Monoclonal/63-1171-82
Sca1-PE-Cy7: https://www.thermofisher.cn/cn/zh/antibody/product/Ly-6A-E-Sca-1-Antibody-clone-D7-Monoclonal/25-5981-82
CD150-FITC: https://www.thermofisher.cn/cn/zh/antibody/product/CD150-Antibody-clone-mShad150-Monoclonal/11-1502-82
CD48-APC: https://www.thermofisher.cn/cn/zh/antibody/product/CD48-Antibody-clone-HM48-1-Monoclonal/17-0481-82
B220-FITC: https://www.thermofisher.cn/cn/zh/antibody/product/CD45R-B220-Antibody-clone-RA3-6B2-Monoclonal/11-0452-82
CD19-APC-e780: https://www.thermofisher.cn/cn/zh/antibody/product/CD19-Antibody-clone-eBio1D3-1D3-Monoclonal/47-0193-82
IgM-APC: https://www.thermofisher.cn/cn/zh/antibody/product/IgM-Antibody-clone-II-41-Monoclonal/17-5790-82
CD43-PE: https://www.thermofisher.cn/cn/zh/antibody/product/CD43-Antibody-clone-eBioR2-60-Monoclonal/12-0431-82
CD24-APC-e780: https://www.thermofisher.cn/cn/zh/antibody/product/CD24-Antibody-clone-M1-69-Monoclonal/47-0242-82
CD71-FITC: https://www.thermofisher.cn/cn/zh/antibody/product/CD71-Transferrin-Receptor-Antibody-clone-R17217-RI7-217-1-4-Monoclonal/11-0711-82
Ter119-PE: https://www.thermofisher.cn/cn/zh/antibody/product/TER-119-Antibody-clone-TER-119-Monoclonal/12-5921-82
CD11b-FITC: https://www.thermofisher.cn/cn/zh/antibody/product/CD11b-Antibody-clone-M1-70-Monoclonal/11-0112-82
Gr1-PE: https://www.thermofisher.cn/cn/zh/antibody/product/Ly-6G-Antibody-clone-1A8-Ly6g-Monoclonal/12-9668-82
Ultra-LEAF™ Purified anti-mouse CD3ε Antibody: https://www.biolegend.com/en-us/products/ultra-leaf-purified-anti-mouse-cd3epsilon-antibody-7722
Ultra-LEAF™ Purified anti-mouse CD28 Antibody: https://www.biolegend.com/en-us/products/ultra-leaf-purified-anti-mouse-cd28-antibody-7733
TCRβ-PE: https://www.thermofisher.cn/cn/zh/antibody/product/TCR-beta-Antibody-clone-H57-597-Monoclonal/12-5961-82

Animals and other organisms

Policy information about studies involving animals; ARRIVE guidelines recommended for reporting animal research

Laboratory animals

Tox4 floxed mice were generated by CRISRP-Cas9 mediated knock-in of floxed exons 4-6 into fertilized eggs from mice of C57BL/6 background in collaboration with Shanghai Model Organisms Center, Inc. Lck-Cre mice were purchased from Shanghai Model Organisms Center, Inc.

Wild animals

No wild animals were used in this study.

Field-collected samples

No field-collected samples were used in this study.

Ethics oversight

Mice were bred and maintained in pathogen-free facilities at Shanghai Jiao Tong University and studies were conducted in accordance with the Shanghai Jiao Tong University Animal Studies Committee Regulations.

Note that full information on the approval of the study protocol must also be provided in the manuscript.

Flow Cytometry

Plots

Confirm that:

- [x] The axis labels state the marker and fluorochrome used (e.g. CD4-FITC).
- [x] The axis scales are clearly visible. Include numbers along axes only for bottom left plot of group (a 'group' is an analysis of identical markers).
- [x] All plots are contour plots with outliers or pseudocolor plots.
- [x] A numerical value for number of cells or percentage (with statistics) is provided.

Methodology

Sample preparation	Thymocytes and lymphocytes were washed and resuspended in PBS plus 1% fetal bovine serum (FBS) at a concentration of 1-5 x 106/ml. All the staining were performed on ice unless stated otherwise. For surface protein staining, cells were stained with anti-CD16/32 antibodies for 10 min to block non-specific binding to Fc receptors before the stain with antibodies targeting surface proteins of interest. Intracellular staining for Ki67 was performed using the Foxp3/Transcription factor staining Buffer Set (eBioscience, cat. no. 00-5523-00). For Annexin V staining, cells were rested in RPMI-1640 supplemented with 10% FBS for 5 hrs at 37°C before stained with an anti-Annexin antibody in Binding Buffer (0.01 M HEPES, 0.14 M NaCl, and 2.5 mM CaCl2). Dead cells were excluded by 7-Aminoactinomycin D (7-AAD) staining.
Instrument	All the flow cytometry analyses were performed on a Beckman Coulter CytoFLEX flow cytometer. The sorting of DP thymocytes was performed on a BD FACSAria III sorter.
Software	All flow cytometry data was analyzed using CytExpert version 2.3.0.84.
Cell population abundance	Abundance is reported in figures where relevant. Sorting was performed with "Purity" settings, thus purity is >95%.
Gating strategy	Gating was determined using fluorescent-minus-one controls for each color used in each FACS experiment to ensure that positive populations were solely associated with the antibody for that specific marker.

- [x] Tick this box to confirm that a figure exemplifying the gating strategy is provided in the Supplementary Information.
